# Supplementary material for: Circulatory miRNA as a Biomarker for Therapy Response and Disease-Free Survival in Hepatocellular Carcinoma
Source: Cancers (Basel). 2020 Sep 29;12(10):2810. doi: 10.3390/cancers12102810 (PMC7601056; doi:10.3390/cancers12102810)
Supplement: Supplementary file 1 [file cancers-12-02810-s001.pdf]

cancers

Supplementary Materials: Circulatory miRNA as a Biomarker for Therapy Response and Disease-Free Survival in Hepatocellular Carcinoma

Table S1. Demographic characteristics of the study populations.

	Patients	Curative treatment		TACE		
	n = 86	CR *n* = 27	PRPD *n* = 13	CR *n* = 14	PRPD *n* = 32	***p value****
**Age (mean, 95%CI)**	72.4 (70.8-74.0)	71.8 (69.1-74.5)	74.3 (69.4-79.1)	74.4 (68.8-80)	69.6 (66.7-72.5)	ns
**Sex (M/F)**	69/17	21/6	10/3	8/6	30/2	*P<0.01*
**Etiology**						
Alcohol metabolic	46	12	7	7	20	*ns*
Alcohol metabolic viral	14	5	3	3	3	
Viral	20	7	3	3	7	
Other**	5	2	0	1	2	
**Disease scores**						
CTP A/B	64/19	20/6	12/1	12/2	20/10	*ns*
BCLC 0A/BC	61/24	26/1	12/1	9/5	14/17	*P<0.001*
**Number of lesions**						
Single < 2cm	10	4	2	2	2	*ns*
Single or 3 ≤3cm	45	19	8	6	12	
Large-single or multi	30	4	3	6	17	
**Alpha fetoprotein**						
<20 ng/mL	49	16	7	10	16	*ns*
20 - 400 ng/mL	12	2	3	1	6	
>400 ng/mL	6	2	1		3	

**p* value considers the difference between clinical parameters with therapy response (CR and PRPD) considering all type of treatments (curative and non-curative); ** autoimmune, hemochromatosis, cryptogenic, usually in combination with other.

Table S2. Average miRNA expression according to microarray profiling analysis between CR and PRPD in HCC samples. CR = Complete responder, PRPD = Partial responder and progressive diseases.

miRNA	Log 2 Avg Sig. (95%CI) in CR	Log 2 Avg Sig. (95%CI) in PRPD	FC (linear) CR vs. PRPD	*p* Value
miR-4454	3.98 (3.05–4.90)	1.80 (1.28–2.31)	4.89	0.003
miR-4443	2.17 (1.62–2.72)	4.23 (2.92–5.74)	0.26	0.009
miR-1275	1.39 (0.94–1.84)	2.18 (1.27–3.09)	0.41	0.013
miR-4492	3.12 (2.02–4.21)	1.56 (2.22–5.62)	4.00	0.017
miR-885-3p	1.3 (0.98–1.74)	1.86 (1.11–2.60)	0.49	0.030
miR-4722-3p	1.99 (1.64–2.34)	1.48 (1.30–1.67)	1.81	0.031
miR-2116-3p	1.16 (1.03–1.29)	1.42 (1.16–1.69)	0.67	0.037
miR-4439	1.15 (1.03–1.29)	1.29 (1.16–1.41)	0.79	0.037
miR-4530	3.89 (2.62–5.06)	2.28 (1.43–3.12)	2.92	0.045

Table S3. Differently expressed miRNA candidates at T0, according to response to treatment. Data are shown as mean expression (95% CI). CR = Complete responder, PRPD = Partial responder and progressive diseases.

	T0		
**miRNA**	**CR (95% CI)**	**PRPD (95% CI)**	***p* value**
**miR-4443**	2.7(1.86-3.54)	5.14 (3.21-7.07)	0.089
**miR-4454**	0.32 (0.22-0.43)	0.17 (0.11-0.23)	0.010**
**miR-4492**	21.86 (15.55-28.16)	11.84 (9.10-14.57)	0.006***
**miR-4530**	791.90 (52.60-1057.00)	555.42 (289.4-821.15)	0.015*

© 2020 by the author. Licensee MDPI, Basel, Switzerland. This article is an open access article distributed under the terms and conditions of the Creative Commons Attribution (CC BY) license (http://creativecommons.org/licenses/by/4.0/).
